# First Melody® valve implantations in Africa

**DOI:** 10.5830/CVJA-2015-007

**Published:** 2015

**Authors:** DG Buys, C Greig, SC Brown

**Affiliations:** Department of Paediatric Cardiology, University of the Free State, and Universitas Hospital, Bloemfontein, South Africa; Department of Paediatric Cardiology, University of the Free State, and Universitas Hospital, Bloemfontein, South Africa; Department of Paediatric Cardiology, University of the Free State, and Universitas Hospital, Bloemfontein, South Africa

**Keywords:** Melody® valve, Africa, percutaneous valve, implantation

## Abstract

Congenital heart lesions involving the right ventricular outflow tract (RVOT) are a common problem in paediatric cardiology. These patients need multiple surgical interventions in the form of valved conduits over a lifetime. Surgical re-valvulation was the standard treatment option until the introduction of percutaneous pulmonary valves over a decade ago. These valves can be used to prolong the lifespan of conduits and reduce the number of re-operations. The Melody® valve (Medtronic, Minneapolis, MN, USA) was introduced as the first dedicated percutaneous pulmonary valve. Percutaneous pulmonary valves can be implanted successfully and have the advantage of short hospitalisations. We describe the first three Melody® valve implantations in Africa.

## Abstract

Right ventricle-to-pulmonary artery (RV–PA) conduit failure is a vexing problem in post-operative congenital cardiac lesions involving the right ventricular outflow tract (RVOT). Typical lesions include tetralogy of Fallot, pulmonary atresia, truncus arteriosus, and others. These lesions often require early intervention and multiple RVOT revisions. Surgical re-interventions may result in prolonged hospital stay with increased morbidity and mortality rates.^1^,^2^ Due to the invasive nature of surgery, some patients with RVOT dysfunction are managed for years before surgical re-valvulation is considered.

The first percutaneous pulmonary valve was implanted in the year 2000, and led to the development of the Melody® valve (Medtronic, Minneapolis, MN, USA).^3^-^5^ The Melody® valve consists of an 18-mm valve segment, the Contegra® modified bovine jugular vein, sutured into a platinum iridium stent of 34-mm length (Fig. 1). The valve can be crimped down to 6 mm and re-expanded from 18 to 22 mm using the Ensemble® transcatheter delivery system (Medtronic, Minneapolis, MN, USA).

**Figure 1.**
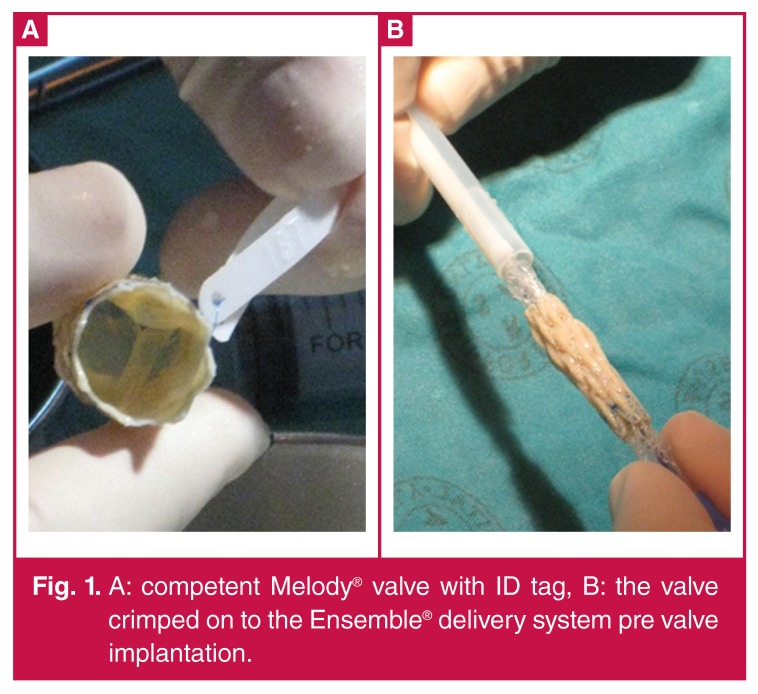
A: competent Melody® valve with ID tag, B: the valve crimped on to the Ensemble® delivery system pre valve implantation.

We describe the first three Melody® valve implantations in Africa. These were done at the Universitas Academic Hospital complex in Bloemfontein, South Africa.

## Case report

**Figure 2.**
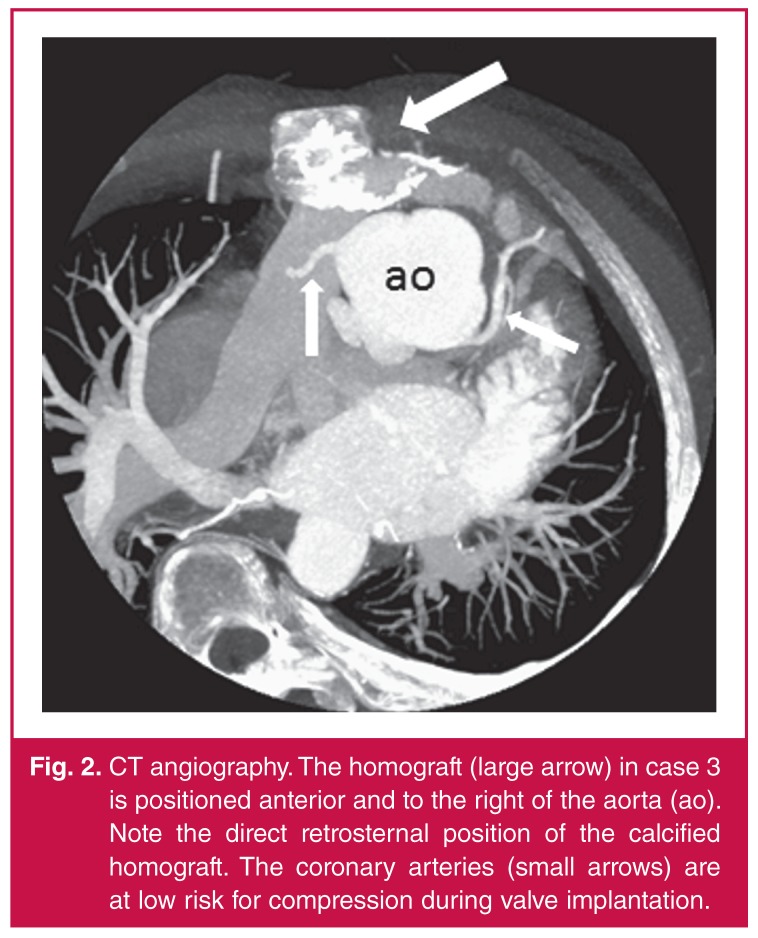
CT angiography. The homograft (large arrow) in case 3 is positioned anterior and to the right of the aorta (ao). Note the direct retrosternal position of the calcified homograft. The coronary arteries (small arrows) are at low risk for compression during valve implantation.

Only patients meeting standard indications for surgical re-intervention were evaluated for transcatheter valve implantation. Extensive work up included: chest radiography, electrocardiography (ECG), evaluation of exercise capacity, echocardiography, and high-resolution computed tomographic angiography (CTA) (Fig. 2). The right ventricle size and function as well as the severity of pulmonary regurgitation (PR) and/or pulmonary stenosis (PS) were assessed and quantified. CTA in all three patients demonstrated favourable coronary artery anatomy and we proceeded with valve implantation in March 2012.

## Case 1

The patient was a 17-year-old male (weight 46.2 kg) with tetralogy of Fallot. As initial intervention, he had had surgical correction and a RVOT patch at 18 months of age. This was later followed by a RV-PA outflow tract reconstruction and insertion of a 20-mm homograft at age 11 years. He was considered for percutaneous valve implantation because of exercise intolerance, right ventricular dysfunction and severe PR.

The echocardiogram demonstrated a severely dilated right ventricle, free PR and a RVOT peak instantaneous gradient (PIG) of 60 mmHg. After successful pre-stenting of the RVOT, a Melody® valve was successfully implanted using a 22-mm Ensemble® system (see Methods). No residual PS or PR was demonstrated post valve implantation.

## Case 2

The second case was a 16-year-old female (weight 50.0 kg) with double-outlet right ventricle (DORV) and pulmonary stenosis. She had had a DORV correction and a 20-mm aortic homograft insertion at the age of 12 years. RVOT rehabilitation was indicated due to a mixed lesion of PS and PR.

Echocardiography demonstrated a calcified RVOT with a PIG of 52 mmHg and a dilated right ventricle with moderate to severe PR. Coronary artery anatomy was favourable. A 22-mm Ensemble® delivery system was used to successfully implant a Melody® valve (see Methods). The right ventricle pressure was reduced with no residual PR.

## Case 3

**Figure 3.**
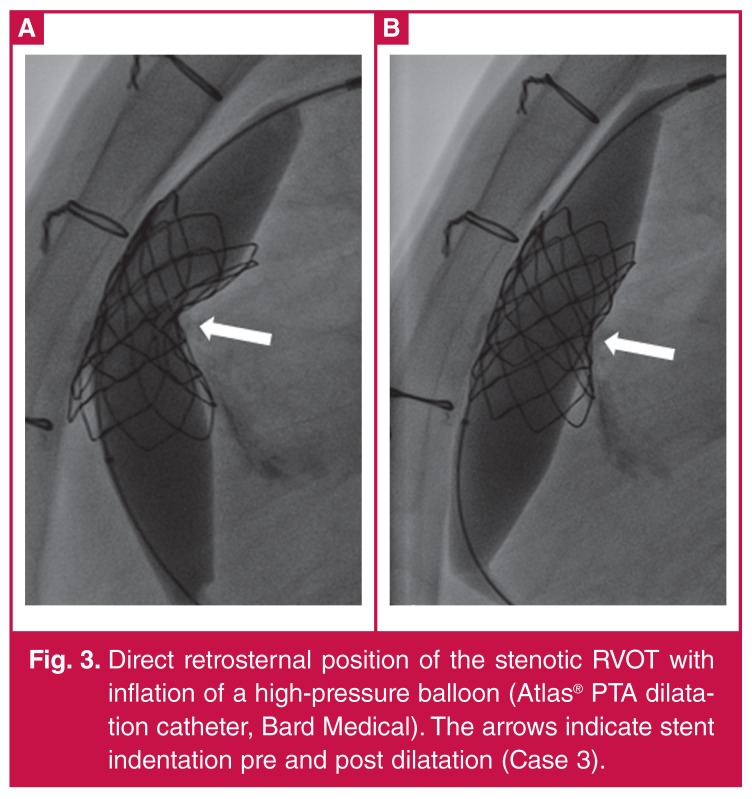
Direct retrosternal position of the stenotic RVOT with inflation of a high-pressure balloon (Atlas® PTA dilatation catheter, Bard Medical). The arrows indicate stent indentation pre and post dilatation (Case 3).

The patient was a 31-year-old male (weight 47.5 kg) with DORV and pulmonary stenosis. He had had RVOT reconstruction with a 21-mm homograft at age 17 years. Surgery was considered risky due to a direct retrosternal location of the original homograft (Fig. 2, Fig. 3). His main indication for re-valvulation was pulmonary stenosis with a RVOT PIG of 60 mmHg.

A stable ‘landing site’ was constructed using two stents and pre-dilation with a high-pressure balloon (see Methods). The Melody® valve was then delivered using an 18-mm Ensemble® delivery system. The valve was dilated using a high-pressure balloon and a good result was achieved. The patient did well with mild transient retrosternal chest pain as the only complaint.

## Methods

Valve implantation was performed under general anaesthesia. All patients were heparanised following our standard protocol, and prophylactic antibiotics (Cefazolin) were given. Vascular access was obtained via the femoral vessels and haemodynamic data were collected pre and post Melody® valve implantation (Table 1).

**Table 1:** Haemodynamic information

	*RV pressure (mmHg)*		*MPA pressure (mmHg)*		*PR*	
	*pre*	*post*	*pre*	*post*	*pre*	*post*
Case 1	35/6	32/6	20/7	20/8	severe	none
Case 2	74/6	42/6	52/13	32/17	severe	none
Case 3	56/6	34/6	24/6	24/9	moderate	none

RV: right ventricle; MPA: main pulmonary artery pressure; PR: pulmonary regurgitation.

**Figure 4.**
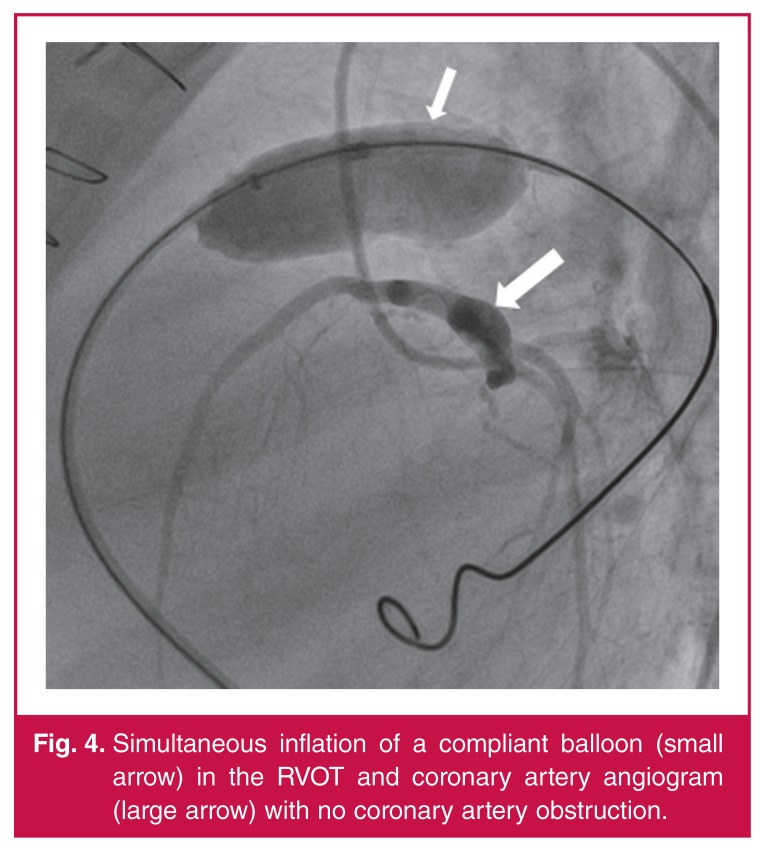
Simultaneous inflation of a compliant balloon (small arrow) in the RVOT and coronary artery angiogram (large arrow) with no coronary artery obstruction.

Simultaneous coronary angiography and inflation of a low-pressure balloon (Amplatzer^TM^ sizing balloon II, St Jude Medical, St Paul, MN, USA) in the RVOT was performed to exclude coronary artery occlusion (Fig. 4).There were no coronary artery occlusions or ECG changes detected during balloon inflation, and the decision was made to continue with the procedure.

Preparing the landing zone for Melody® valve implantation is a crucial step in the procedure. It is important to record any stent recoil during balloon deflation. A stiff guide wire [Meier (Boston Scientific, Natick, MA, USA), LunderquistTM extra stiff (Cook Medical, Bloomington, USA)] with stable position was obtained. In our experience, pre-stenting of the RVOT is more challenging and once the landing site is prepared, the Melody® valve is implanted with minimal difficulty.

Pre-dilation and pre-stenting was performed in all three cases. This was achieved using BIB® (NuMED, Hopkins, NY, USA) balloons and bare-metal stents (IntraStentTM LD MaxTM, ev3 Endovascular, Plymouth, USA) in cases 1 and 2. Both patients needed only one pre-stent to obtain a stable RVOT with no recoil of stent during balloon deflation and no residual stenosis.

Case 3 was more complicated due to the direct retrosternal position of the RVOT. The RVOT was severely calcified and residual stenosis and recoil of a 45-mm covered CP stent (NuMED, Hopkinton, NY, USA) using a 22-mm BIB® warranted a second stent implantation to secure a stable landing zone. This was achieved using a 36-mm IntraStent^TM^ LD Max^TM^ (ev3 Endovascular, Plymouth, USA) on a 22-mm BIB®. Residual indentation of the stents was abolished using a high-pressure balloon (Bard Atlas® PTA dilatation catheter, Bard Peripheral Vascular, Tempe, AZ, USA) (Fig. 3).

**Figure 5.**
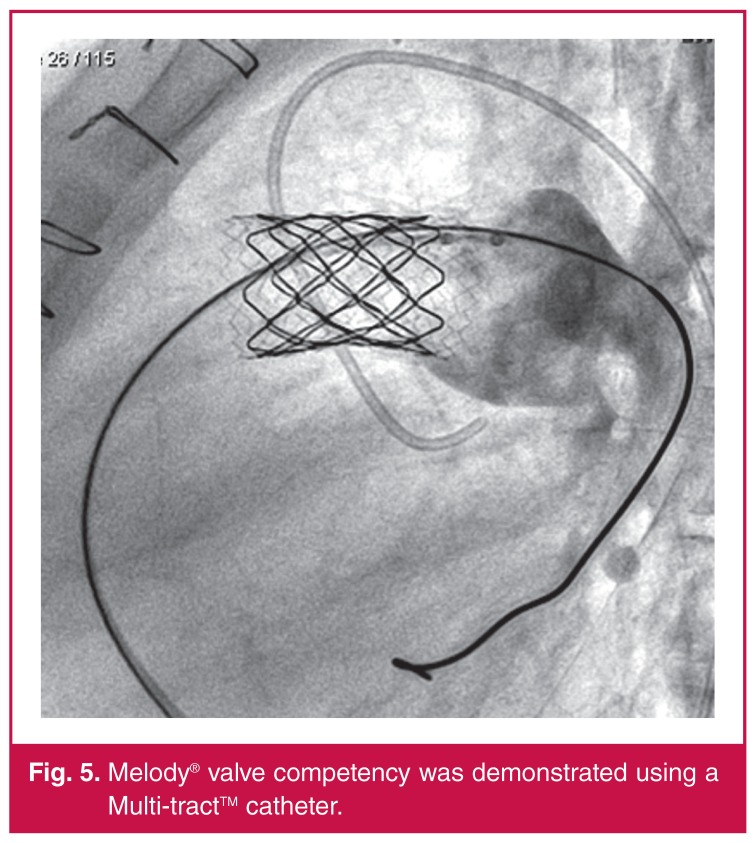
Melody® valve competency was demonstrated using a Multi-tract^TM^ catheter.

Once RVOT rehabilitation was completed, the Melody® valves were successfully implanted. Patient 3 needed post-valve implant dilatation due to the residual stenosis and gradient. Post-stent implantation gradients were measured using a Multitrack^TM^ angiographic catheter (NuMED, Hopkinton, NY, USA). At the time of implantation, the decision was made that the results were satisfactory and no further dilatation was indicated. Melody® valve implantation was successful in all three patients, with reduction of RVOT gradients and elimination of the PR (Fig. 5).

Post valve implantation coronary angiograms were normal with no vessel obstruction. There were no ischaemic changes on ECG for 48 hours and no pericardial effusion on echocardiography. The patients were observed overnight and discharged home within 48 hours.

The patients have now been followed up for two years and demonstrated RV size and function improvements with normal pulmonary valve function. Subjectively all patients reported improved exercise tolerance.

## Discussion

Percutaneous pulmonary valve implantation has become an accepted alternative to surgical pulmonary re-valvulation, with low morbidity and mortality rates.^4^,^5^ More than 7 000 Melody® valves have been implanted in 156 centres worldwide, and these are the first cases in Africa (direct correspondence with Medtronic).

Indications for percutaneous valve implantation are identical to surgical indications. Classic indications for Melody® valve implantation include: patients above 20 kg, conduit dysfunction with stenosis or moderate regurgitation, conduit size > 16 mm and < 22 mm, and favourable RVOT morphology.^4^-^8^ Contraindications consist of active endocarditis and a conduit size that is incompatible with the valve size.^4^,^7^

Helpful information obtained from CTA includes the anatomical aspects of the RVOT and its spatial relationship to the coronary arteries. The risk of coronary artery compression is the most frequent exclusion factor and cause of procedurerelated deaths. Major procedural complications include dislodgement of the valve, coronary artery compression, rupture of the homograft, and haemothorax due to pulmonary artery perforation. Follow-up complications include stent fracture and endocarditis.^4^-^6^,^8^,^9^ Stent fracture rates diminished after the practice of presenting became common place.

Case 3 demonstrates that percutaneous valve implantation may be a useful alternative to surgery. The transient chest pain in this patient was secondary to RVOT stretching and similar to that of surgery. The benefits of percutaneous valve implantation include short hospital stay and no ICU care. The availability of these valves may reduce the duration of RV dysfunction and the total number of RV–PA conduit replacements.

## Conclusion

Introduction of the Melody® valve has been proven a safe and effective alternative to surgery for RVOT re-valvulation. The Melody® valve can be implanted with a high success rate and low morbidity and mortality rates. Our cases show that a percutaneous valve is a reasonable alternative to surgical re-valvulation in developing countries due to short hospital stay and reduced re-operation rates.

## References

[1] Gober V, Berdat P, Pavlovic M (2005). Adverse mid-term outcome following RVOT reconstruction using the Contegra valved bovine jugular vein.. Ann Thorac Surg.

[2] Sugita T, Ueda Y, Matsumoto M (2000). Repeated procedure after radical surgery for tetralogy of Fallot.. Ann Thorac Surg.

[3] Bonhoeffer P, Boudjemline Y, Saliba Z (2000). Percutaneous replacement of pulmonary valve in a right-ventricle to pulmonary-artery prosthetic conduit with valve dysfunction. Lancet.

[4] Lurz P, Coats L, Khambadkone S (2008). Percutaneous pulmonary valve implantation: impact of evolving technology and learning curve on clinical outcome.. Circulation.

[5] McElhinney DB, Hellebrand WE, Zahn EM (2010). Short- and mediumterm outcomes after transcatheter pulmonary valve placement in the expanded multicenter US Melody valve trail.. Circulation.

[6] Khambadkone S, Coats L, Taylor A (2005). Percutaneous pulmonary valve implantation in humans: results in 59 consecutive patients.. Circulation.

[7] Zahn EM, Hellenbrand WE, Lock JE (2009). Implantation of the melody transcatheter pulmonary valve in patients with a dysfunctional right ventricular outflow conduit: early results from the U.S. clinical trial.. J Am Coll Cardiol.

[8] Eichen A, Ewert P, Hager A (2011). Percutaneous pulmonary valve implantation: two-center experience with more than 100 patients.. Eur Heart J.

[9] Guccione P, Gagliardi MG, Calcagni G (2009). Percutaneous implantation of pulmonary valves for treatment of right ventricular outflow tract dysfunction: a single-centre experience.. J Pead.

